# Pulsed field ablation for atrial fibrillation: clinical applications and research advances

**DOI:** 10.3389/fcvm.2025.1679578

**Published:** 2025-09-23

**Authors:** Meng Sun, XiaoHong Fu, Jia Gao, Min Guo, Wenjie Yin, Rui Wang

**Affiliations:** Department of Cardiology, First Hospital of Shanxi Medical University, Taiyuan, China

**Keywords:** atrial fibrillation, pulsed field ablation, irreversible electroporation, pulmonary vein isolation, myocardial selectivity, safety, emerging technologies

## Abstract

Atrial fibrillation (AF) is the most common sustained cardiac arrhythmia, associated with increased risks of stroke, heart failure, and mortality. With the advancement of catheter ablation technology, pulsed field ablation (PFA), a novel nonthermal ablation modality, has garnered growing attention due to its myocardial selectivity and favorable safety profile. This review systematically summarizes the biophysical principles, clinical advantages, catheter systems, special population applications, limitations, and future directions of PFA based on the latest evidence.

## 1 Introduction

Atrial fibrillation (AF) is the most prevalent sustained arrhythmia worldwide, with an estimated global burden exceeding 100 million by 2050 (1). Catheter ablation has become a cornerstone of rhythm control, with pulmonary vein isolation (PVI) as its fundamental strategy (2). While traditional thermal techniques such as radiofrequency ablation (RFA) and cryoablation are effective, their limitations are associated with heat-related complications (3). PFA, with its nonthermal mechanism, myocardial specificity, and procedural efficiency, is emerging as a new standard in AF ablation (4). As AF continues to increase in both incidence and complexity, there is a growing need for safer and more effective ablation technologies (5).

## 2 Biophysical mechanism

PFA is based on the principle of irreversible electroporation (IRE), delivering high-voltage, short-duration electrical pulses (*μ*s to ms scale) to create permanent nanoscale pores in the cell membrane, leading to apoptotic rather than necrotic cell death (6, 7). This form of programmed cell death minimizes inflammatory response and scar formation. Due to cell-specific electroporation thresholds, cardiomyocytes are particularly susceptible to this energy, enabling myocardial selectivity while sparing adjacent structures such as nerves and vasculature. Recent animal and ex vivo studies have shown that electroporation causes predictable lesion geometry, with sharp borders and minimal collateral thermal spread (8, 9). In contrast to thermal injury, which often leads to heterogeneous tissue damage, PFA lesions exhibit low levels of fibrotic remodeling and preserve surrounding extracellular matrix integrity (10). In porcine and canine models, histological evaluation at 30–90 days post-PFA demonstrated preserved esophageal architecture and coronary artery integrity (11). Furthermore, biphasic waveform pulses have been shown to reduce muscle contraction during energy delivery, thereby improving catheter stability (12). Computational modeling of electric field distributions has demonstrated that tissue thickness, orientation of fibers, and electrode design strongly influence ablation efficacy. Emerging designs such as adjustable lasso- or flower-petal–shaped arrays are intended to optimize lesion uniformity across varying pulmonary vein anatomies (13).

Compared to traditional thermal methods, these characteristics position PFA as a potentially safer and more targeted ablation modality.

A comparative summary is shown in Table 1.

**Table 1:** Provides a direct comparison between PFA and conventional thermal ablation techniques, summarizing differences in mechanisms, safety, and procedural characteristics.

Parameter	Thermal ablation (RF/Cryo)	Pulsed field ablation (PFA)
Ablation mechanism (15, 40)	Heat/Cold-induced necrosis	Nonthermal electroporation
Tissue specificity (15, 40)	Low (non-selective to myocardium)	High (myocardial-selective)
Collateral damage risk (15, 40)	Higher	Lower (tissue selective)
Procedure time (25)	Longer (120-180 min typical)	Shorter (60–90 min typical)
Fluoroscopy requirement (25)	Moderate	Lower (towards zero-fluoroscopy)
Esophageal injury (7, 41)	Common concern	Rare
Phrenic Nerve palsy (7, 41)	Possible	Very Rare
Pulmonary vein stenosis (7, 41)	Reported	Rare
Pain level (15, 41)	Moderate–High	Minimal (biphasic)
Lesion predictability (25)	Variable	High (sharp margins, uniform)

## 3 Clinical advantages

PFA has shown several advantages over thermal ablation:

(1) Safety: The MANIFEST-17 K registry involving over 17,000 patients reported a major complication rate <1%, with rare esophageal injury, pulmonary vein stenosis, or phrenic nerve palsy (14).
(2) Procedural Efficiency: Multi-electrode pentaspline or balloon-in-basket catheters enable single-shot PVI, significantly reducing ablation time. In real-world settings, median procedure durations have been reduced to under 90 min (15). Different PFA catheter systems have been developed to meet procedural demands.

A comparison of Representative PFA Catheter Systems in Table 2.

(3) Myocardial Selectivity: PFA spares surrounding vascular, neural, and esophageal tissues and does not induce chronic fibrosis, offering potential for better long-term outcomes (10). Comparative analysis with thermal methods suggests that PFA nearly eliminates the risk of atrio-esophageal fistula—a catastrophic complication associated with RF (15). Additionally, the risk of thrombus formation appears lower due to the nonthermal mechanism, reducing anticoagulation-related concerns (16). The capacity to achieve wide-area circumferential ablation without the need for lesion dragging is particularly advantageous in reproducibility and safety.

**Table 2:** Summarizes the key features, advantages, and limitations of representative systems.

Catheter type	Representative system	Key features	Advantages	Limitation
Pentaspline (5, 15)	Farawave (Boston Scientific)	Five-spline flexible lattice; Single-shot PVI	Rapid deployment, reproducible lesion set	Wide-field delivery may trigger coronary vasospasm
Balloon-in-basket (42)	Affera Sphere-9 (Medtronic)	Spherical mapping-ablation integration; adjustable energy levels	combined mapping & ablation; good catheter stability	Anatomical adaptability limited, especially in variant PVs
Spiral (36)	VARIPULSE (Biosense Webster)	Helical electrode array; segmental control for selective targeting	Navigable in complex anatomy; precision segmental lesions	Requires high operator experience; longer procedure time
Hexaspline (Novel) (43)	HexaPulse (Emerging systems)	Six-leaf flower configuration; enhanced vein wall contact	Uniform energy distribution; adaptable to PV anatomy	Limited clinical data; not widely avilable

Notably, the admIRE pivotal trial, a large-scale multicenter study, demonstrated the safety and effectiveness of the VARIPULSE™ system in treating paroxysmal atrial fibrillation, reporting a 75% overall primary effectiveness rate and a low 2.9% major complication rate, with no device- or procedure-related deaths or atrioesophageal fistulas. Acute procedural success was achieved in all patients, with 98% first-pass isolation per vein, and 43% of patients discharged on the same day (17).

## 4 Pulmonary vein isolation outcomes

The pivotal ADVENT trial demonstrated that PFA achieved noninferior 12-month freedom from atrial arrhythmia (70.9%) compared to RFA or cryoablation, with fewer complications (5). Additional trials such as PULSE-EU (16) and IMPULSE/PEFCAT (15) confirmed high acute PVI success and lesion durability. The PFA approach also exhibited reduced fluoroscopy times and minimal edema formation on cardiac MRI post-ablation (14). In many studies, remapping after 3 months revealed over 90% durable PVI in initial sites, which compares favorably with rates observed after conventional thermal ablation (15, 17).

Studies such as ADVENT and PULSE-EU have reported approximately 70%–88% freedom from AF at 12 months. In addition, the admIRE pivotal trial offers further insight into acute success and short-term lesion durability. The study demonstrated 85% peak primary effectiveness in patients receiving 73–96 applications, reinforcing the procedural consistency and predictable lesion formation of PFA (17).

Notably, lesion contiguity and transmurality are enhanced due to the uniformity of electric field application, even in regions with complex geometry.

To facilitate cross-study comparison, we summarize procedural success rates, complication incidences, and arrhythmia-free survival from major PFA clinical trials in Table 3. Despite inherent differences in study design, follow-up duration, and patient populations, this compilation offers a quasi-quantitative perspective on the overall performance of PFA.

**Table 3:** Summary of major PFA trials.

Study	Population	Type	Enrollment(n)	Follow-up (months)	PVI Success Rate (%)	Freedom from AF (%)	Major Complication Rate (%)
ADVENT (5)	Paroxysmal AF	RCT, Prospective, Randomized, single-blind, multicenter noninferiority trial	607	12	100	73.3	2.1
PULSE-EU (16)	Paroxysmal & Persistent AF	First-in-human, Prospective, Multi-center, single-arm trial	48	12	100	84.2(P), 80(Per)	2.1
IMPULSE (15)	Paroxysmal AF	Prospective, multicenter, non-randomized, first-in-human feasibility studies	121	12	100	78.5/84.5 (optimized group)	2.5
ADVANTAGE AF (18)	Persistent AF	Prospective,multicenter,single-arm study	255	12	100	78.1	1.3
MANIFEST-PF (37)	Paroxysmal & Persistent AF	Prospective,multicenter,real-world registry	1,568	12	99.2	78.1	1.9
PLEASE-AF (22)	Paroxysmal AF	Prospective,multicenter,single-arm study	143	12	100	86.7	0.7
AdmIRE (17)	Paroxysmal AF	Pivotal, multicenter, prospective trial	277	12	100	75.4	2.9
inspIRE (38)	Paroxysmal AF	Prospective, multicenter, single-arm study	186	12	97.1	75.6(PEE), 81.7(symptomatic), 85.8(real-world estimate)	0 (Wave II )
EU-PORIA (39)	Paroxysmal & Persistent AF	Prospective, multicenter registry, real-world	1,233	12	99–100	74	1.7

AF, atrial fibrillatioN; PVI, pulmonary vein isolation; RCT, randomized controlled trial; P, paroxysmal; Pers, persistent.

A summary of key clinical trial outcomes is provided in Table 3 for comparative reference.

## 5 Beyond PVI applications

Beyond pulmonary vein isolation (PVI), PFA has been increasingly utilized in adjunctive ablation strategies such as posterior wall isolation (PWI), left atrial appendage isolation (LAAI), and hybrid surgical ablation. In the ADVANTAGE AF trial, the addition of PWI using PFA in patients with persistent atrial fibrillation resulted in a 78.1% freedom from atrial arrhythmia at 12 months, with a low major complication rate of 1.3% (*N* = 160) (18). Early clinical experience also supports the feasibility of intracardiac echocardiography (ICE)-guided superior vena cava (SVC) isolation using PFA, offering precise lesion delivery while minimizing collateral damage. The tissue selectivity and safety profile of PFA further make it an attractive candidate for hybrid ablation approaches, particularly in patients with complex substrates or anatomical variations (19).

## 6 Persistent AF and special populations

In patients with persistent AF (PerAF), the ADVANTAGE AF study reported promising outcomes using PFA with additional posterior wall ablation (PVI + PWA), showing reduced atrial injury and potential preservation of atrial contractility (18). Posterior wall substrate modification has been increasingly recognized for its role in PerAF maintenance. The nonthermal nature of PFA allows for linear ablation along the posterior wall without thermal stacking or excessive collateral injury. Moreover, PFA holds specific value in populations with limited procedural reserve:

- For elderly patients (≥75 years), safety and efficacy were comparable to younger cohorts. This is significant given the increasing frailty and polypharmacy in aging populations (20).
- In heart failure (HF) patients, the MANIFEST-PF registry showed improved LVEF and sinus rhythm maintenance post-PFA. Nonthermal ablation may reduce myocardial edema and inflammation, benefiting those with impaired diastolic function (21).
- The PLEASE-AF study confirmed comparable efficacy and safety in Asian populations, highlighting the global applicability of this technique (22).
- In patients undergoing repeat ablation, the safety and feasibility of PFA were supported by the multicenter MANIFEST-REDO study, which included 427 redo procedures for AF or atrial tachycardia recurrences (23). Importantly, in a propensity-matched analysis, FARAPULSE™ demonstrated a significantly lower pulmonary vein reconnection rate (19.1%) compared with cryoballoon (27.5%) or radiofrequency ablation (34.8%), suggesting enhanced lesion durability in redo settings (24).
- Beyond prospective multicenter trials, large-scale real-world data from the MANIFEST-17 K registry—which encompassed 17,642 patients across 106 centers—further substantiated the favorable safety profile of PFA. The study reported a major complication rate of 0.98%, with no procedure-related deaths or atrioesophageal fistula. Notably, sub-analyses of patients undergoing repeat ablation confirmed the safety and feasibility of PFA in both index and redo procedures, emphasizing its versatility in clinical practice (14).

## 7 Lesion durability and long-term outcomes

In addressing persistent AF, the ADVANTAGE AF Phase 2 trial pioneered the use of continuous implantable cardiac monitoring in evaluating PFA efficacy. Among 255 cohort patients undergoing PVI plus posterior wall ablation (with 55.3% also receiving CTI ablation), freedom from atrial arrhythmias at 12 months was 73.4%, with a low major adverse event rate of 2.4%. Detailed monitoring revealed that 52% of patients had no arrhythmic recurrence, and 94% had no episode lasting more than 24 h. Under stringent AA burden thresholds (≤0.1%) and episode duration (<1 h), 12-month procedural effectiveness rates were 71.6% and 70.0%, respectively. These findings support PFA's safety and efficacy in treating persistent AF with superior arrhythmia suppression and healthcare utility outcomes (18).

While most current studies focus on acute efficacy and short-term follow-up, long-term safety and effectiveness data are emerging. In the pivotal admIRE US IDE trial, the VARIPULSE™ system demonstrated a primary effectiveness of 74.6% at 12 months, with a major adverse event rate of 2.9%.These findings highlight the durable lesion formation and favorable safety profile of PFA in a multicenter, prospective setting (17).

Long-term follow-up from early feasibility trials provides encouraging durability data. In fact, 116 patients (95.9% retention) from the IMPULSE/PEFCAT series demonstrated a sustained arrhythmia-free survival of 73.3% at a median 49-month follow-up (25).

## 8 Limitations and challenges

Despite the promising advantages, PFA adoption faces several limitations:

(1) Lack of full integration with advanced electroanatomic mapping systems restricts its utility in complex arrhythmias such as atrial tachycardias or atypical flutters (17).
(2) Coronary vasospasm has been reported, especially with wide-field pentaspline catheters, requiring nitroglycerin pretreatment. Procedural workflows may require adaptation in patients with known coronary artery disease (26).
(3) PFA's minimal autonomic denervation may limit its adjunctive effects in modulating AF substrate in certain patient groups (27). Further studies are needed to quantify neural impacts. In addition, the cost of PFA equipment and disposables remains high, which may limit adoption in resource-limited settings until local manufacturing or reimbursement pathways are established.
(4) PFA has shown not only clinical efficacy but also procedural and economic advantages. In the admIRE trial, the VARIPULSE™ system demonstrated high procedural efficiency, with a median procedure time of 81 min and fluoroscopy time of just 7 min. Notably, 25% of procedures were completed without fluoroscopy due to CARTO™ 3 system integration, and 43% of patients were discharged on the same day (28). These operational metrics suggest improved workflow and potential cost savings. Complementing these observations, recent economic modeling indicates that while the initial cost of PFA systems is higher than that of RFA or cryoballoon, per-patient savings may range from €511 to €1,497 when accounting for reduced resource utilization and procedure times (29).

While early evidence suggests that PFA may reduce procedural time and hospital resource use, comprehensive health economics studies—including cost-effectiveness modeling and long-term outcome analyses from ongoing trials such as ADVENT extension and ADMIRE—are needed to validate these advantages.

## 9 Evidence appraisal and limitations

While growing clinical evidence continues to support the safety and efficacy of PFA, the interpretation of these findings must be approached with caution. Notably, considerable heterogeneity exists across studies in terms of design (e.g., observational vs. prospective trials), patient selection criteria, and procedural protocols (22). For instance, the ADVENT trial focused solely on paroxysmal AF patients, whereas studies like ADVANTAGE-AF and MANIFEST-PF included cohorts with persistent AF (18, 20). Differences in procedural techniques, operator experience, and outcome definitions further complicate direct comparisons across trials (18, 22). Additionally, the involvement of device manufacturers in several major studies may introduce bias in trial design and reporting (20, 22). These factors may limit the external validity of current findings and underscore the need for independent, multicenter investigations with standardized protocols and long-term follow-up to verify the durability and reproducibility of PFA outcomes (20, 22).

## 10 Future perspectives

Future directions for PFA include:

- Development of nanosecond PFA (nsPFA) for enhanced precision and reduced energy exposure (30);
- Integration of contact force sensing, real-time lesion assessment, and 3D mapping for individualized lesion delivery (31);
- Expansion into other arrhythmias: Early animal data suggest feasibility of PFA in ventricular tachycardia and cavotricuspid isthmus ablation (32);
- Potential application in left atrial appendage isolation, posterior wall homogenization, and hybrid surgical approaches (33, 34);
- Use in patients with heart failure with preserved ejection fraction (HFpEF), who may benefit from reduced ablation-induced scarring (21);
- Continued development of zero-fluoroscopy workflows to reduce radiation exposure (29);
- Domestic device development and healthcare policy support for cost control and broader access (28, 35).

In January 2024, Biosense Webster announced the regulatory approval of the VARIPULSE^TM^ pulsed field ablation (PFA) system in Japan, marking the first CARTO-integrated PFA system to receive such authorization in the country. This milestone reflects growing global momentum toward adopting catheter platforms with full electroanatomical mapping compatibility for enhanced procedural safety and efficiency (36).

In the latest registry evaluation of the VARIPULSE™ PFA platform, an overall 75% primary effectiveness rate was reported, with peak efficacy reaching 85% among patients receiving 73–96 applications. The system demonstrated a favorable safety profile, with a low adverse event rate (2.9%) and no device- or procedure-related deaths or major complications. Notably, acute success was achieved in all patients, and 98% first-pass isolation was recorded per vein. The median procedure time was 81 min for PVI-only cases, with a fluoroscopy time of 7 min. Impressively, 43% of patients were discharged the same day, and 25% of procedures were performed fluoroless without compromising outcomes, enabled by full integration with the CARTO™ 3 mapping system (17).

### 10.1 Future directions and optimization strategies

Pulsed-field ablation (PFA) near coronary arteries—especially using wide-field pentaspline catheters—has been shown to provoke coronary vasospasm in a significant proportion of cases, raising safety concerns. However, recent evidence supports the use of high-dose parenteral nitroglycerin pre-treatment as an effective prophylactic measure, significantly reducing the incidence of severe vasospasm during PFA delivery (26).

In parallel, broader clinical adoption of PFA depends not only on its safety and efficacy but also on practical implementation factors. Prioritizing the development of domestically produced PFA systems and improving integration with widely used electroanatomic mapping platforms (e.g., CARTO, EnSite) may reduce dependency on expensive imported devices and streamline procedural workflows.

Although economic evaluations of PFA remain limited, such technical and infrastructural advancements are expected to enhance cost-effectiveness and accessibility, particularly in low- and middle-income healthcare settings, thus supporting the global scalability of PFA technology (17, 28, 35).

## 11 Conclusion

PFA represents a paradigm shift in AF catheter ablation, combining myocardial selectivity, procedural efficiency, and improved safety. Current data support its noninferiority in paroxysmal AF and its applicability in persistent AF and high-risk populations. As technology matures and long-term evidence accumulates, PFA is poised to become a frontline ablation modality, enabling more patients to benefit from this safe and effective treatment. Continued innovation in device design and procedural integration will determine the pace of its global adoption.

## References

[1] Chugh SS Havmoeller R Narayanan K Singh D Rienstra M Benjamin EJ Worldwide epidemiology of atrial fibrillation: a global burden of disease 2010 study. Circulation. (2014) 129(8):837–47. 10.1161/CIRCULATIONAHA.113.005119 24345399 PMC4151302

[2] Bunch TJ Cutler MJ. Is pulmonary vein isolation still the cornerstone in atrial fibrillation ablation? J Thorac Dis. (2015) 7(2):132–41. 10.3978/j.issn.2072-1439.2014.12.46 25713728 PMC4321067

[3] Cappato R Calkins H Chen SA Davies W Iesaka Y Kalman J Updated worldwide survey on the methods, efficacy, and safety of catheter ablation for human atrial fibrillation. Circ Arrhythm Electrophysiol. (2010) 3(1):32–8. 10.1161/CIRCEP.109.859116 19995881

[4] Chun KJ Miklavčič D Vlachos K Bordignon S Scherr D Jais P State-of-the-art pulsed field ablation for cardiac arrhythmias: ongoing evolution and future perspective. Europace. (2024) 26(6):euae134. 10.1093/europace/euae134 Erratum in: Europace. 2024 July 2;26(7):euae182. doi: 10.1093/europace/euae182. 38848447 PMC11160504

[5] Reddy VY Gerstenfeld EP Natale A Whang W Cuoco FA Patel C Pulsed field or conventional thermal ablation for paroxysmal atrial fibrillation. N Engl J Med. (2023) 389(18):1660–71. 10.1056/NEJMoa2307291 37634148

[6] Sullivan AP Aguilar M Laksman Z. Pulsed field ablation: a review of preclinical and clinical studies. Bioengineering (Basel). (2025) 12(4):329. 10.3390/bioengineering12040329 40281689 PMC12024434

[7] Hartl S Reinsch N Füting A Neven K. Pearls and pitfalls of pulsed field ablation. Korean Circ J. (2023) 53(5):273–93. 10.4070/kcj.2023.0023 37161743 PMC10172271

[8] Miklavčič D Verma A Krahn PRP Štublar J Kos B Escartin T Biophysics and electrophysiology of pulsed field ablation in normal and infarcted porcine cardiac ventricular tissue. Sci Rep. (2024) 14(1):32063. 10.1038/s41598-024-83683-y 39738639 PMC11686391

[9] Gómez-Barea M García-Sánchez T Ivorra A. A computational comparison of radiofrequency and pulsed field ablation in terms of lesion morphology in the cardiac chamber. Sci Rep. (2022) 12(1):16144. 10.1038/s41598-022-20212-9 36167959 PMC9515184

[10] Neven K van Es R van Driel V van Wessel H Fidder H Vink A Acute and long-term effects of full-power electroporation ablation directly on the porcine esophagus. Circ Arrhythm Electrophysiol. (2017) 10(5):e004672. 10.1161/CIRCEP.116.004672 28487347

[11] Hsu JC Gibson D Banker R Doshi SK Gidney B Gomez T *In vivo* porcine characterization of atrial lesion safety and efficacy utilizing a circular pulsed-field ablation catheter including assessment of collateral damage to adjacent tissue in supratherapeutic ablation applications. J Cardiovasc Electrophysiol. (2022) 33(7):1480–8. 10.1111/jce.15522 35510408 PMC9545022

[12] Bi S Jia F Lv C He Q Xu X Xue Z Preclinical study of biphasic asymmetric pulsed field ablation. Front Cardiovasc Med. (2022) 9:859480. 10.3389/fcvm.2022.859480 35402543 PMC8987372

[13] Ezzeddine FM Asirvatham SJ Nguyen DT. Pulsed field ablation: a comprehensive update. J Clin Med. (2024) 13(17):5191. 10.3390/jcm13175191 39274404 PMC11396515

[14] Ekanem E Neuzil P Reichlin T Kautzner J van der Voort P Jais P Safety of pulsed field ablation in more than 17,000 patients with atrial fibrillation in the MANIFEST-17K study. Nat Med. (2024) 30(7):2020–9. 10.1038/s41591-024-03114-3 38977913 PMC11271404

[15] Reddy VY Dukkipati SR Neuzil P Anic A Petru J Funasako M Pulsed field ablation of paroxysmal atrial fibrillation: 1-year outcomes of IMPULSE, PEFCAT, and PEFCAT II. JACC Clin Electrophysiol. (2021) 7(5):614–27. 10.1016/j.jacep.2021.02.014 33933412

[16] Turagam MK Neuzil P Petru J Funasako M Koruth JS Skoda J AF Ablation using a novel “single-shot” map-and-ablate spherical array pulsed field ablation catheter: 1-year outcomes of the first-in-human PULSE-EU trial. Heart Rhythm. (2024) 21(8):1218–26. 10.1016/j.hrthm.2024.04.102 38768840

[17] Reddy VY Calkins H Mansour M Wazni O Di Biase L Bahu M AdmIRE trial investigators. Pulsed field ablation to treat paroxysmal atrial fibrillation: safety and effectiveness in the AdmIRE pivotal trial. Circulation. (2024) 150(15):1174–86. 10.1161/CIRCULATIONAHA.124.070333 39258362 PMC11458102

[18] Reddy VY Gerstenfeld EP Schmidt B Andrade JG Nair D Natale A Pulsed field ablation of persistent atrial fibrillation with continuous electrocardiographic monitoring follow-up: aDVANTAGE AF phase 2. Circulation. (2025) 152(1):27–40. 10.1161/CIRCULATIONAHA.125.074485 40273320 PMC12225731

[19] Pierucci N La Fazia VM Mohanty S Schiavone M Doty B Gabrah K Results of ICE-guided isolation of the superior vena Cava with pulsed field ablation. JACC Clin Electrophysiol. (2025) 11(4):752–60. 10.1016/j.jacep.2024.11.009 39846925

[20] Turagam MK Neuzil P Schmidt B Reichlin T Neven K Metzner A Impact of left atrial posterior wall ablation during pulsed-field ablation for persistent atrial fibrillation. JACC Clin Electrophysiol. (2024) 10(5):900–12. 10.1016/j.jacep.2024.01.017 38430087

[21] Turagam MK Neuzil P Schmidt B Reichlin T Neven K Metzner A Safety and effectiveness of pulsed field ablation for atrial fibrillation in patients with heart failure: a MANIFEST-PF sub-analysis. JACC Clin Electrophysiol. (2024) S2405-500X(24):00351–7. 10.1016/j.jacep.2024.05.002 38864809

[22] Wang Z Tang M Reddy VY Chu H Liu X Xue Y Efficacy and safety of a novel hexaspline pulsed field ablation system in patients with paroxysmal atrial fibrillation: the PLEASE-AF study. Europace. (2024) 26(7):euae174. 10.1093/europace/euae174 38912887 PMC11223653

[23] Scherr D Turagam MK Maury P Blaauw Y van der Voort P Neuzil P Repeat procedures after pulsed field ablation for atrial fibrillation: mANIFEST-REDO study. Europace. (2025) 27(8):euaf012. 10.1093/europace/euaf012 39824172 PMC12344414

[24] Della Rocca DG Marcon L Magnocavallo M Menè R Pannone L Mohanty S Pulsed electric field, cryoballoon, and radiofrequency for paroxysmal atrial fibrillation ablation: a propensity score-matched comparison. Europace. (2023) 26(1):euae016. 10.1093/europace/euae016 38245007 PMC10823352

[25] Yavin H Prasad M Gordon J Aksu T Huang HD. Contemporary trends in pulsed field ablation for cardiac arrhythmias. J Cardiovasc Dev Dis. (2024) 12(1):10. 10.3390/jcdd12010010 39852288 PMC11766314

[26] Malyshev Y Neuzil P Petru J Funasako M Hala P Kopriva K Nitroglycerin to ameliorate coronary artery spasm during focal pulsed-field ablation for atrial fibrillation. JACC Clin Electrophysiol. (2024) 10(5):885–96. 10.1016/j.jacep.2023.12.015 38385916

[27] Tohoku S Schmidt B Schaack D Bordignon S Hirokami J Chen S Impact of pulsed-field ablation on intrinsic cardiac autonomic nervous system after pulmonary vein isolation. JACC Clin Electrophysiol. (2023) 9(9):1864–75. 10.1016/j.jacep.2023.05.035 37480870

[28] Calvert P Mills MT Xydis P Essa H Ding WY Koniari I Cost, efficiency, and outcomes of pulsed field ablation vs thermal ablation for atrial fibrillation: a real-world study. Heart Rhythm. (2024) 21(9):1537–44. 10.1016/j.hrthm.2024.05.032 38763378

[29] Hirata S Nagashima K Watanabe R Wakamatsu Y Hirata M Kurokawa S Workflow of the zero-fluoro pulsed field ablation. J Arrhythm. (2024) 40(6):1529–32. 10.1002/joa3.13174 39669927 PMC11632259

[30] Nies M Watanabe K Kawamura I Wang BJ Litt J Turovskiy R Ablating myocardium using nanosecond pulsed electric fields: preclinical assessment of feasibility, safety, and durability. Circ Arrhythm Electrophysiol. (2024) 17(7):e012854. 10.1161/CIRCEP.124.012854 38758741 PMC11254255

[31] Anić A Phlips T Brešković T Mediratta V Girouard S Jurišić Z Pulsed field ablation using focal contact force-sensing catheters for treatment of atrial fibrillation: 1-year outcomes of the ECLIPSE AF study. Circ Arrhythm Electrophysiol. (2025) 18(1):e012794. 10.1161/CIRCEP.124.012794 39698744 PMC11753462

[32] Della Rocca DG Cespón-Fernández M Keelani A Raffa S Pannone L Almorad A Focal pulsed field ablation for premature ventricular contractions: a multicenter experience. Circ Arrhythm Electrophysiol. (2024) 17(9):e012826. 10.1161/CIRCEP.124.012826 39234745

[33] Molitor N Berger F Breitenstein A Badertscher P Kühne M Sticherling C Percutaneous left atrial appendage occluders can impair pulmonary vein isolation by pulsed field ablation. Heart Rhythm. (2025) S1547-5271(25):00207–3. 10.1016/j.hrthm.2025.01.047 40010621

[34] Gunawardene MA Schaeffer BN Jularic M Eickholt C Maurer T Akbulak RÖ Pulsed-field ablation combined with ultrahigh-density mapping in patients undergoing catheter ablation for atrial fibrillation: practical and electrophysiological considerations. J Cardiovasc Electrophysiol. (2022) 33(3):345–56. 10.1111/jce.15349 34978360

[35] Katapadi A Garg J Kabra R Lakkireddy D. High stakes and high voltage: the real costs of pulsed field ablation: a response to “cost, efficiency, and outcomes of pulsed-field ablation vs thermal ablation for atrial fibrillation: a real-world study.”. Heart Rhythm. (2024) S1547-5271(24):03642–7. 10.1016/j.hrthm.2024.05.032

[36] Biosense Webster. Regulatory Approval of VARIPULSE™ PFA System in Japan. (2024). Available at: Available online at: https://www.jnj.com/media-center/press-releases/biosense-webster-announces-regulatory-approval-of-varipulse-pulsed-field-ablation-pfa-platform-in-japan.

[37] Turagam MK Neuzil P Schmidt B Reichlin T Neven K Metzner A Safety and effectiveness of pulsed field ablation to treat atrial fibrillation: one-year outcomes from the MANIFEST-PF registry. Circulation. (2023) 148(1):35–46. 10.1161/CIRCULATIONAHA.123.064959 37199171

[38] De Potter TJR Grimaldi M Duytschaever M Anic A Vijgen J Neuzil P Long-term clinical benefits of pulsed field ablation in paroxysmal atrial fibrillation: subanalyses from the multicenter inspIRE trial. Circ Arrhythm Electrophysiol. (2025) 18(5):e013465. 10.1161/CIRCEP.124.013465 40276859 PMC12094256

[39] Schmidt B Bordignon S Neven K Reichlin T Blaauw Y Hansen J European real-world outcomes with pulsed field ablatiOn in patients with symptomatic atRIAl fibrillation: lessons from the multi-centre EU-PORIA registry. Europace. (2023) 25(7):euad185. 10.1093/europace/euad185 37379528 PMC10320231

[40] Reddy VY Anic A Koruth J Petru J Funasako M Minami K Pulsed field ablation in patients with persistent atrial fibrillation. J Am Coll Cardiol. (2020) 76(9):1068–80. doi: 10.1016/j.jacc.2020.07.007 32854842 10.1016/j.jacc.2020.07.007

[41] Verma A Boersma L Haines DE Natale A Marchlinski FE Sanders P First-in human experience and acute procedural outcomes using a novel pulsed field ablation system: the PULSED AF pilot trial. Circ Arrhythm Electrophysiol. (2022) 15(1):e010168. 10.1161/CIRCEP.121.010168 34964367 PMC8772438

[42] Stewart MT Haines DE Verma A Kirchhof N Barka N GrassI E Intracardic pulsed field ablation: proof of feasibility in a chronic porcine model. Heart Rhythm. (2019) 16(5):754–64. 10.1016/j.hrthm.2018.10.030 30385383

[43] Kueffer T Tanner H Madaffari A Seiler J Haeberlin A Maurhofer J Posterior wall ablation by pulsed-field ablation: procedural safety, efficacy, and findings on redo procedures. Europace. (2023) 26(1):euae006. 10.1093/europace/euae006 38225174 PMC10803044

